# The Failure Mechanical Properties of Cemented Paste Backfill with Recycled Rubber

**DOI:** 10.3390/ma16093302

**Published:** 2023-04-22

**Authors:** Baogui Yang, Xiaolong Wang, Chengjin Gu, Faguang Yang, Hao Liu, Junyu Jin, Yibo Zhou

**Affiliations:** School of Energy and Mining Engineering, China University of Mining and Technology, Beijing 100083, China

**Keywords:** mechanical behavior, uniaxial compressive strength, impact resistance, digital image correlation technique

## Abstract

Understanding the mechanical properties and failure process of cemented paste backfill with recycled rubber (RCPB) is the foundation of backfill design in underground mining. In this study, physical and mechanical tests were conducted on RCPB to obtain its mechanical property parameters, such as its uniaxial compressive strength (UCS), toughness, and peak strain. The influence of the rubber dosage on the mechanical properties of RCPB was also analyzed. In addition, the deformation behavior, fracture development, and failure process of RCPB with different rubber contents were observed using the digital image correlation (DIC) technique. The experimental results suggested that, although the UCS of RCPB is reduced as more rubber is added, its toughness and ability to absorb energy is increased. Moreover, the impact resistance of RCPB is improved by this increased toughness. With the increase in the rubber content, the deformation corresponding to the plastic yield stage of RCPB increased, which resulted in better ductility and improved impact resistance. The failure of the RCPB specimens mainly showed an “X” shape. The results of this study help us to better understand the mechanical behavior of RCPB after backfilling underground.

## 1. Introduction

As a safe and environmentally friendly disposal method for tailings, backfilling has significant advantages in controlling surface deformation and in improving ore recovery. Its benefits for use in underground mines are being recognized increasingly around the world [1,2,3,4,5,6,7]. The materials comprising cemented paste backfill (CPB) are important in maintaining mine safety and stability [8,9,10,11]. Therefore, the mechanical properties of the paste have always represented an important research direction in underground mining [12].

In the process of filling after adjacent ore blasting, the impact generated by the blasts requires the impact resistance of CPB to be high [13,14,15]. If the impact resistance of CPB is weak, it will lose its designed protection function, and it will increase both the dilution rate of the ore and the beneficiation cost because the broken filling body will fall with the ore [16,17,18]. Therefore, improving the impact resistance of filling bodies has always been the main research direction [19]. The improvement of the impact resistance of CPB is mainly achieved by reducing its brittleness and increasing its toughness [20,21,22,23].

To date, many scholars have conducted research on increasing the toughness of CPB. Cao et al. [24] studied the influence of different contents (0%, 3%, 6%, and 9%) of polypropylene, polyacrylonitrile, and glass fiber on the strength and toughness of CPB. It was found that the addition of fibers significantly improved the toughness of CPB. Furthermore, the impact of the different types of fibers on the toughness of CPB was also evaluated. Zhou et al. [25] studied the effect of glass fibers of different lengths (3 mm, 6 mm, and 15 mm) on the mechanical properties of CPB and concluded that the most suitable length was 6 mm. Wang et al. [26] studied the influence of corn stalk fiber on the mechanical properties of CPB. They found that the addition of corn stalk fiber increased the mechanical properties of CPB and that the reinforcement effect of corn stalk fiber after alkaline treatment was better. Li et al. [27] studied the influence of straw fiber on the tensile properties of CPB. It was found that the addition of straw fiber increased the tensile properties of the CPB, and it was also found that this strengthening effect was weakened when the curing time was increased. The above-mentioned research demonstrated that adding substances to CPB increases its toughness [28,29,30,31,32]. However, to date, there have been few studies on the influence of rubber on the mechanical properties of backfill.

With the rapid development of automobiles all over the world, the output of waste rubber is increasing each day. More waste tires are now forming “black pollution”, threatening the living environment of the entire human race [33]. The pollution caused by waste rubber has gradually attracted more attention [34]. Waste rubber has a strong heat resistance and mechanical properties. Therefore, it does not easily degrade naturally; it will not rot and deteriorate if it is abandoned on the Earth’s surface or buried underground for more than ten years [35]. When waste rubber is piled up in the open air for a long time, it will occupy much land space and pollute the soil [36,37]. Although the proportion of waste rubber in solid waste is not high, it is one of the main problems in waste disposal. Consequently, recycling rubber is the most efficient method of dealing with the issue of waste rubber [38].

To date, there have been some studies on the influence of recycled rubber on the mechanical properties of CPB. Wang et al. [39] studied the effect of rubber fiber on the toughness of CPB and found that, although the rubber fiber (16 mm in length) reduced the strength of the CPB, it increased its toughness. They mainly studied rubber fibers with an average length of 16 mm. However, direct research on the impact of rubber particles on the strength properties, toughness, and the other mechanical properties of CPB is still scarce; therefore, this needs to be further explored.

The strength of CPB mainly comes from the hydration products of cement [40]. When the paste slurry is transported to the area to be filled, the filling slurry gradually solidifies and hardens, with the aid of complex hydration. This leads to the gradual generation of strength [41]. According to its specific application, the CPB must have certain strength requirements to maintain its stability and play a supporting role [18,42,43]. Past studies on the mechanical properties of CPB have focused on its strength and deformation behavior [44]. Uniaxial compressive strength (UCS) is a commonly used mechanical index, which directly reflects the hardening process of CPB [45,46,47,48]. In a UCS test, the CPB can generate, propagate, locate, and accumulate cracks, resulting in a series of stress–strain behaviors [49,50,51]. The strength and deformation behavior are the criteria used to measure the fracture evolution or failure of CPB, and thus, they have been the main focus of CPB research to date.

The failure progress of CPB has been studied using different techniques. The ultrasonic pulse velocity (UPV) method has been effectively used to estimate the strength of CPB and to explore its failure evolution during loading [52,53,54,55]. In recent years, digital image correlation (DIC) techniques have become popular in the mechanical studies of concrete, rock, and other materials. This is a no-touch optical measurement method with a simple operation. The DIC technique can both obtain the full-field strain distribution and characterize the cracking behavior of materials [16,56]. The development of the surface strain field has been successfully monitored by DIC technology. It can be used to explore the fracture evolution process, and it can also be used to both explore and elucidate fracture mechanisms [57].

At present, there has been no direct study into the influence of rubber particles on CPB. Therefore, in this paper, we outline a new method of improving the impact resistance of CPB and for the treatment of waste rubber. This method was determined through our study into the influence of rubber particles on the toughness of CPB. The UPV and UCS tests were used to measure the physical and mechanical properties of the RCPB specimens in this study. The strength and deformation characteristics of RCPB specimens with different rubber contents were also studied. In this study, the toughness of RCPB was explored, and the DIC technique was used to monitor the full field strain of RCPB in the UCS test in real time. To determine the correlation between the deformation behavior and failure evolution of RCPB, its failure progress was explored by visually observing the initiation and propagation of cracks on the specimens’ surfaces.

## 2. Experimental Program

### 2.1. Materials

The tailings used in the experiments were obtained from the underground polymetallic solid mine in Neves-Corvo, Portugal. The tailings were used as the aggregate. Tailings were collected in disc filters at the CPB plant and transported in drums to the Geomechanics Laboratory (GEOLAB) of the Institute for Advanced Study in Lisbon, Portugal, for research.

In this study, 42.5 R-type Portland cement was used as a cementing agent. The cement was supplied by Somincor, from Louléand, and was sold and shipped by Cimpor-Cimentos de Portugal, SGPS, SA.

**Figure 1:**
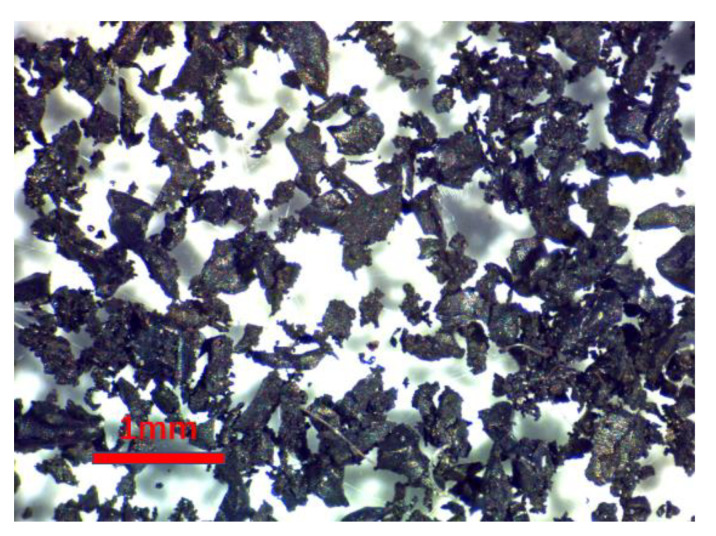
Detailed view of rubber powder under stereomicroscopy observation.

Recycled rubber powder was 100% vulcanized tire rubber powder, supplied by Biogoma -Sociedade Reciclagem de Pneus, Lda. The particle size of rubber powder observed under a stereomicroscope is shown in Figure 1. The main component was irregular, horn-shaped dark particles.

To better simulate the conditions of an engineering site, the water used was industrial water, transported from a mine to the laboratory.

### 2.2. Experimental Design

The purpose of this experiment was to investigate the UCS of RCPB. The paste concentration was maintained at 73.8%, the cement concentration was maintained at 5% of the solid content, and the varied rubber contents were 0%, 1%, 3%, 4%, 5%, and 7% of the solid content. The material mix is shown in Table 1. The calculation of material content is shown in Formulas (1)–(5).

(1) $$\mathrm{Rubber}\;\mathrm{dosage}\;\left(\%\right)=\left(M_{\mathrm{rubber}}/M_{\mathrm{solids}}\right)\times 100$$

(2) $$\mathrm{Cement}\;\mathrm{dosage}\;\left(\%\right)=\left(M_{\mathrm{cement}}/M_{\mathrm{solids}}\right)\times 100$$

(3) $$\mathrm{Tailings}\;\mathrm{dosage}\;\left(\%\right)=\left(M_{\mathrm{tailings}}/M_{\mathrm{solids}}\right)\times 100$$

(4) $$\mathrm{Water}\;\mathrm{dosage}\;\left(\%\right)=\left\{M_{\mathrm{water}}/(M_{\mathrm{water}}+M_{\mathrm{solids}})\right\}\times 100$$

(5) $$M_{\mathrm{solids}}=M_{\mathrm{cement}}+M_{\mathrm{rubber}}+M_{\mathrm{tailings}}$$

where $M_{\mathrm{cement}}$, $M_{\mathrm{rubber}}$, $M_{\mathrm{tailings}}$, $M_{\mathrm{water}}$, and $M_{\mathrm{solids}}$ are the mass of cement, rubber, tailings, water, and solids, respectively (kg).

### 2.3. Specimen Preparation

The RCPB had a cement content of 5% and a solids content of 73.8%. The mixing of the slurry was divided into the following steps:

1. The raw materials were weighed according to the design table, see Table 1;
2. The solid material was poured into the stirring container and stirred for 2 min at a speed of 75 r/min;
3. Water was poured into the stirring container and stirred for 5 min at a speed of 75 r/min.

**Figure 2:**
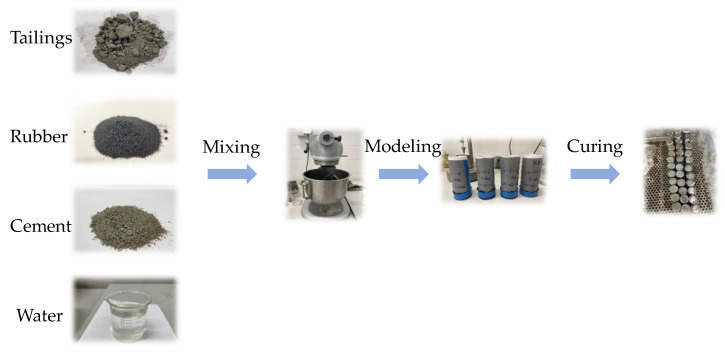
The specimen preparation process.

The slurry was then poured into a standard, cylindrical mold, with a diameter of 46 mm and a height of 100 mm, for UCS testing. The DIC test specimens were made by using a triple test mold, with a size of 70.7 mm × 70.7 mm × 70.7 mm (length × width × height). To simulate the underground paste environment, the specimens were cured in a humidity chamber (YH-40B), which was at (25 ± 2) °C and 85% RH. After reaching the specified curing time (7 d and 28 d), the specimens were taken out for testing. Three specimens were tested in each batch. The preparation process of the specimens is shown in Figure 2.

### 2.4. Methods

To obtain the physical and mechanical properties of specimens with different rubber contents, an ultrasonic longitudinal wave velocity test and a UCS test were carried out.

#### 2.4.1. UCS Test

**Figure 3:**
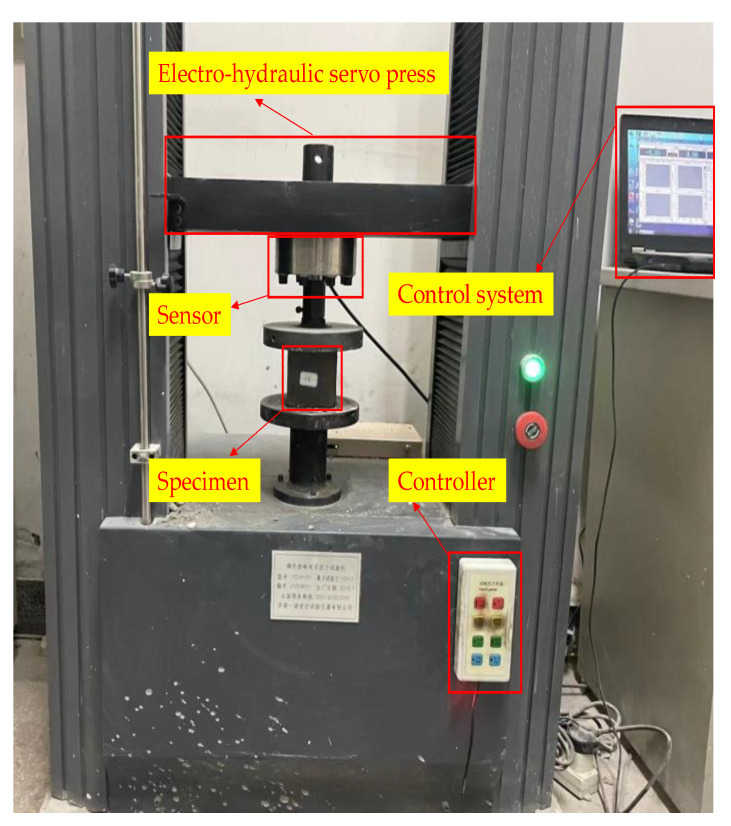
UCS test (TYE-50) equipment.

The UCS test was carried out using the Instron Universal Testing Machine (TYE-50). The equipment is shown in Figure 3. The UCS of the specimens was tested according to the Chinese standard, JGJ/T 70-2009. Before the UCS test was conducted, the dimensions of the specimens were corrected to obtain an effective compression surface. The UCS test was performed using a computer-controlled mechanical press, with a load capacity of 50 kN.

#### 2.4.2. UPV Test

**Figure 4:**
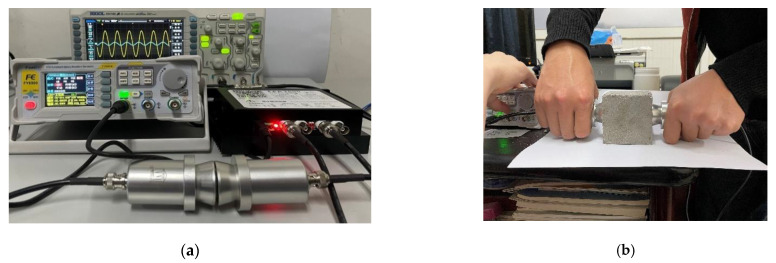
(**a**) Ultrasonic test instruments; (**b**) ultrasonic test.

A non-metallic ultrasonic detector (ZT802) was used to detect ultrasonic pulse velocity. The instrument included a host, signal line, and transducer. The host and sensor were connected by signal lines. The sensor frequency was 50 kHz and the sampling period was 0.4 μs. To ensure complete contact between the sensor surface and the specimen surface, petroleum jelly was applied to the surfaces of the sensors (transmitter and receiver) before testing. The measurement was repeated three times along the axis of the specimen, and the average value of the three tests was taken for analysis. The ultrasonic testing equipment is shown in Figure 4.

#### 2.4.3. DIC Monitoring

**Figure 5:**
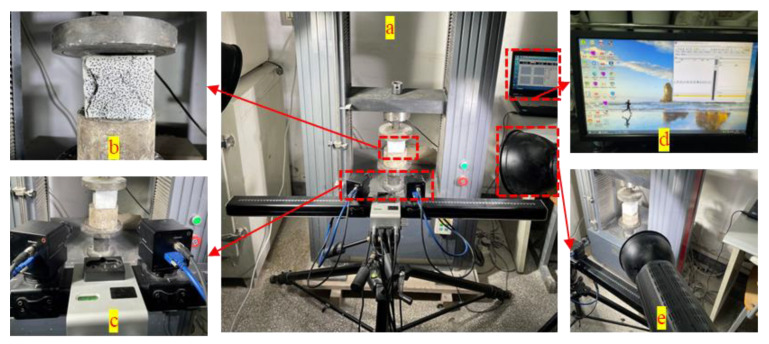
DIC test system: (**a**) Loading system; (**b**) test sample; (**c**) cameras; (**d**) DIC computer; (**e**) light sources.

The DIC technique was applied to observe the surface deformation of each specimen during the UCS tests. The basic principle of the DIC technique is based on tracking the spatial variation in the gray value between the original image and the deformed image. The surface of the specimen was sprayed with white matt paint. The spots were marked with black markers to provide sufficient contrast after the white matt paint dried. Before testing, we took an initial image as a reference for the undeformed specimen. We then took pictures with a camera (RICOH FL-CC1614A-2M), at a speed of 1 s^−1^, to capture deformed images of spots, until the specimen failed. The deformation information of the specimen surface was calculated by referring to the initial digital image and the image of the subsequent deformation state, based on the built-in image processing algorithm of DIC. Figure 5 shows the main parts of the DIC test setup.

## 3. Results

### 3.1. The UCS and UPV

**Figure 6:**
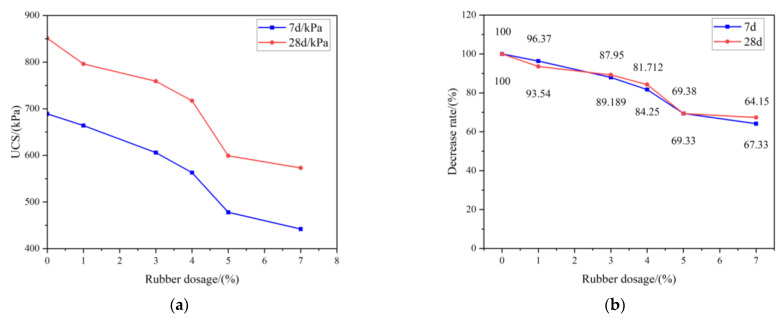
(**a**) The results of UCS tests; (**b**) decrease rate of UCS.

The UCS of RCPB is one of the most important parameters used to characterize its mechanical properties, and, in this study, it was also the main parameter considered for the design of the filling experiment scheme. To explore the relationship between the UCS of the RCPB and the rubber content, we conducted the UCS experiment. First, the slurry was mixed according to the designed ratio and the specimens were prepared. Second, the specimens, which were maintained for 7 and 28 days, were tested using the Instron Universal Testing Machine. The loading rate was 0.2 mm/min. Figure 6a shows the test results.

It can be observed from Figure 6a that the strength of the RCPB decreased as the rubber content was increased. This indicated that the addition of rubber reduces the strength of CPB. This is mainly because the strength of the rubber itself is very low; however, rubber has good toughness and elasticity. Moreover, the interfacial hydrophobicity of rubber causes it to form a layer of water film on its surface when it is mixed with other filling materials to form a slurry. The water film gradually forms pores during the later stage of the solidification process of RCPB. In the end, the original porosity of RCPB is more than that of CPB without rubber. This is the main reason for its decrease in strength.

A rubber content of 0% was used as a control, and R was used to represent the rate of decrease in the strength of the specimen. Through this, the change in the strength of the specimen after the addition of rubber can be seen more clearly. The calculation of R was made using Formula (6), as follows:

(6) $$R=\left(\sigma_{x}/\sigma_{0}\right)\times 100$$

where $\sigma_{x}$ is the peak strength of RCPB, x = 1, 3, 4, 5, and 7. $\sigma_{0}$ is the peak strength of the filling without rubber.

The results are shown in Figure 6b. It is possible to see from this figure that the percentage of strength decreased and that the speed of strength also decreased. We can also see that the strength first decreased slowly, then decreased rapidly, and then decreased slowly again as the rubber content was increased. When the rubber content was increased from 0% to 4%, the strength of the RCPB decreased by 18.3% after 28 days of curing and decreased by 15.7% after 7 days of curing. The strength decreased by more than 30% when the content of rubber was increased to 5%.

**Figure 7:**
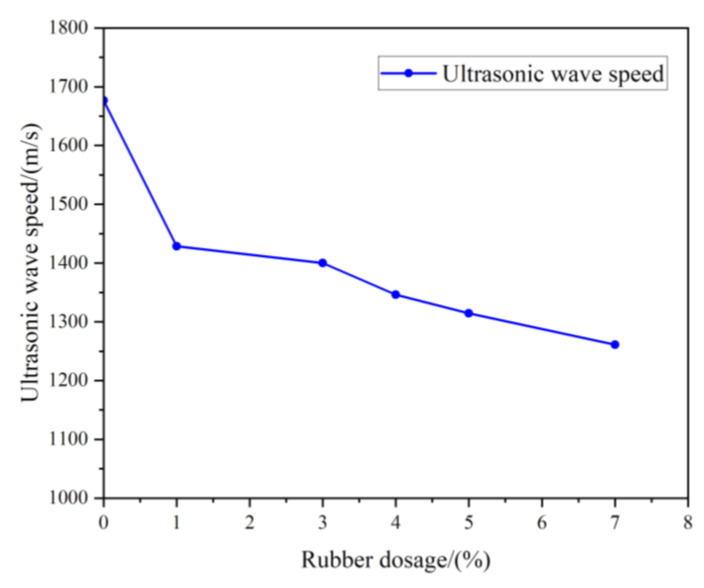
Results of ultrasonic wave velocity test.

The UPV can reflect the structural state inside RCPB. It is one of the most effective indicators for evaluating the failure progress of composite materials. The results of the ultrasonic testing are shown in Figure 7. The propagation speeds of the ultrasonic waves in the RCPB specimens were found by measuring the time required for the ultrasonic waves to penetrate the specimen and by the length of the specimen. It can be seen from Figure 7 that the ultrasonic longitudinal wave velocity of the RCPB specimens decreased with the increase in the rubber content. This is because the propagation speed of ultrasonic waves in rubber is much lower than that of the backfill body—only 80~120 m/s. Therefore, in this study, the UPV in the RCPB decreased with the increase in the rubber content. Furthermore, after the rubber was added to the CPB, the interface formed between the surface of the rubber particles and the RCPB matrix also hindered the propagation of the UPV. When the ultrasonic wave propagates from one medium to another medium of a different density, refraction and reflection occur. This results in weakened energy and a decrease in the wave speed. The relationship between the UPV and RCPB shows the same trend as the relationship between UCS and RCPB, which is gradually reduced.

### 3.2. Peak Strain and Toughness

**Figure 8:**
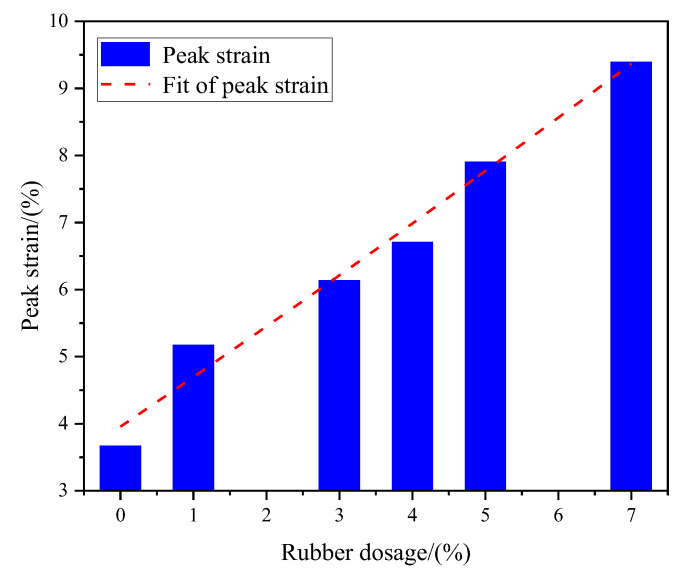
Linear fitting of peak strain and rubber content.

The peak strain of RCPB refers to the corresponding strain when RCPB reaches its peak stress. The RCPB fails when the load on it exceeds its peak stress and the deformation exceeds its peak strain. The peak strain is an important parameter for characterizing the mechanical properties of RCPB, which reflects its deformation ability [58]. The relationship between the peak strain of RCPB and the rubber content is shown in Figure 8. It can be seen from Figure 8 that the greater the rubber content, the greater the peak strain; however, the growth is not unlimited. The relationship between the two was fitted with a quadratic function, and the result was y = 0.0152x^2^ + 0.9617x + 2.9026, and the correlation coefficient, R^2^, was 0.9843. When the rubber content was increased from 0% to 4%, the peak strain of the RCPB increased by 82.5% after 28 days of curing. This means that as the rubber content increases, RCPB has better deformability.

**Figure 9:**
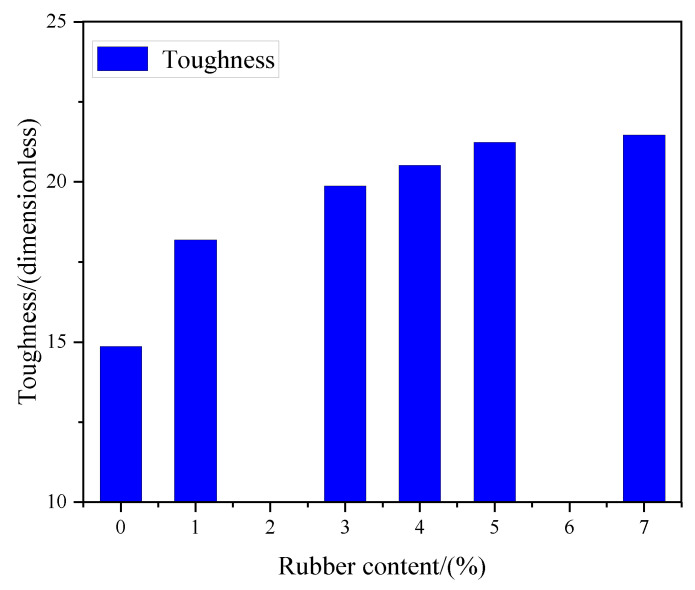
The relationship between the toughness and the rubber content of the RCPB specimens.

The toughness of RCPB reflects its ability to absorb energy and plastic deformation, which is very important for the safety and stability of mining work. According to previous studies, the toughness value of CPB can be determined by calculating the area under the stress–strain curve to the fracture strain. Figure 9 shows the relationship between the toughness and the rubber content of the RCPB specimens. It can be seen from Figure 9 that the toughness of the RCPB increased steadily as the rubber content was increased. In terms of the strength of RCPB, although the rubber caused a reduction in the UCS, it increased its toughness. The rate of increase in its toughness became slower as the rubber content was increased. The toughness of the CPB increased by 38% when the content of rubber was increased from 0% to 4%. This increase in toughness indicated that the ability of CPB to absorb energy was improved by adding rubber. This means that more energy would need to be absorbed to destroy it; therefore, the RCPB had a better energy absorption capacity. These results showed that the impact resistance of CPB is enhanced by recycled rubber.

The reason for this is that the viscoelastic characteristics of the rubber itself endow the filler with better elastic deformation ability. Due to the better deformation ability of rubber, when RCPB reaches its peak strain, it needs to absorb more energy.

### 3.3. Failure Process

**Figure 10:**
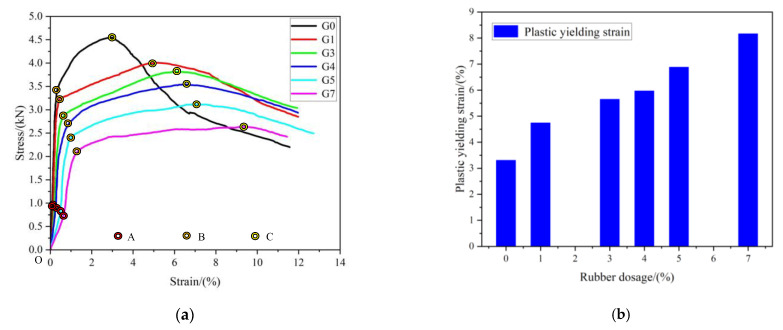
(**a**) Whole-process stress–strain curve of RCPB; (**b**) strain in the plastic-yielding phase.

Figure 10a shows the whole-process stress–strain curve. It can be seen from this figure that the stress–strain curve of RCPB is different from CPB without rubber. This is mainly because the toughness of rubber greatly enhances the ductility of the specimen.

The failure process of RCPB is divided into four main stages: the micropore compaction stage (OA), the linear elastic deformation stage (AB), the plastic yield stage (BC), and the post-peak failure stage (C—). However, the shape of the stress–strain curve of the RCPB also changed under the different rubber contents. It can be seen from Figure 10a that the slope of the straight-line segment of the stress–strain curve of the RCPB showed a decreasing trend when the rubber content was increased. Most obviously, the corresponding strain of segments B and C gradually increased with the increase in the rubber. The residual strength of the RCPB after reaching its peak stress was still high. This means that RCPB maintains high strength after failure.

**Figure 11:**
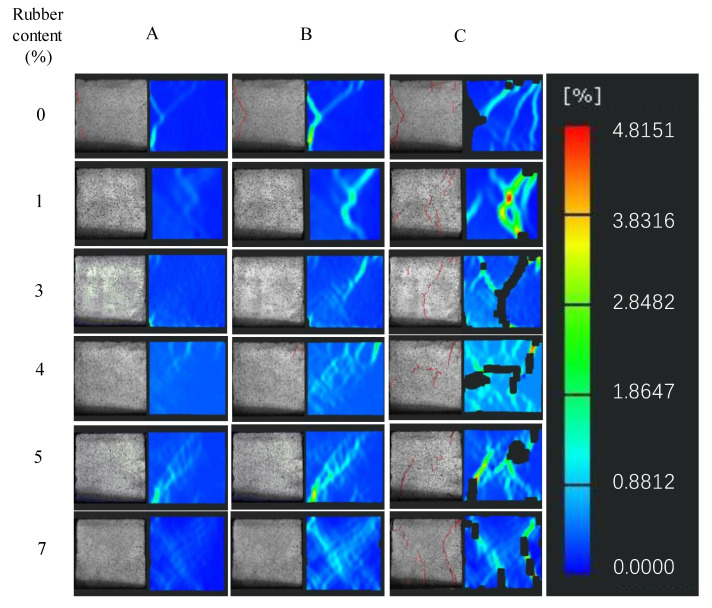
Failure process recorded by the DIC of RCPB specimens with different rubber contents. The (**A**–**C**) in the figure are the beginning of the linear elastic phase, the end of the elastic phase, and the apex of the curve in the stress-strain curve, respectively. The number on the left side of the figure represents the rubber content (%).

Figure 11 shows the failure process recorded by the DIC testing of the RCPB specimens with different rubber contents. It can be seen from Figure 11 that the observed cracks appeared later in the RCPB specimens than they did in the specimen without added rubber. The RCPB corresponds to greater strains at peak failure. No cracks were observed on the surface of the RCPB specimens, as they were on the surface of the specimen without rubber. The deformation of the rubber-free specimen’s surface can be clearly seen from the DIC image. The specimen without rubber was destroyed after reaching its peak stress, and the generation of cracks could be directly observed on its surface. However, the test block did not break directly and had high residual strength. This result was consistent with the result shown in the stress–strain curve.

**Figure 12:**
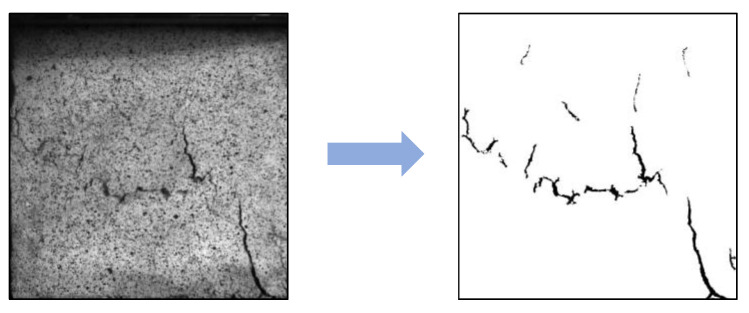
Image binarization processing.

The fractal dimensions of the fissures were used to characterize their development, so that the damage degree of the RCPB could be characterized. The ImageJ software was used to binarize the original image, as shown in Figure 12. The fractal dimensions of the cracks were calculated. The peak strain at the time of the filling bodies’ failures corresponded to the peak strength of the specimens, and the degree of the fractures’ development at this time reflected the residual bearing capacity of the specimens. The effect of rubber on the development and expansion of the RCPB specimens’ cracks was studied.

**Figure 13:**
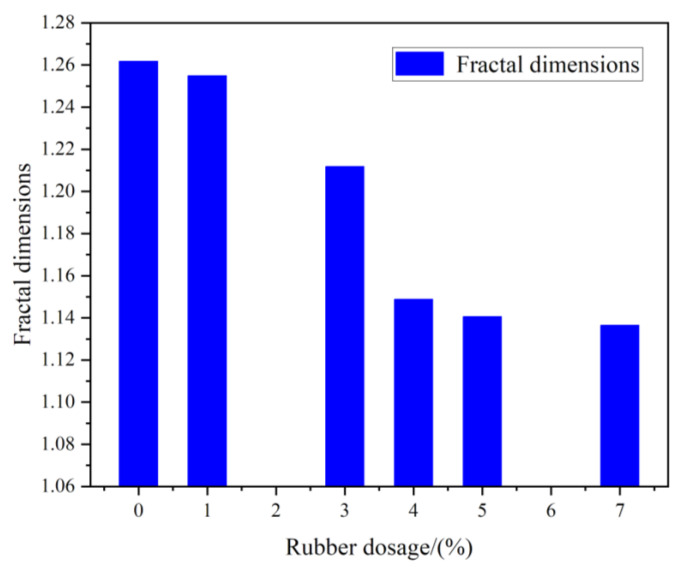
Fractal dimension of samples with different rubber contents after the destruction.

The influence of the rubber on the cracks’ development was characterized by the fractal dimensions. The relationship between the rubber content and the fractal dimensions of the fractures is shown in Figure 13. According to the results, the greater the rubber content, the smaller the degree of the crack development when RCPB reaches its peak strain. This means that the integrity of RCPB is better than that of CPB without rubber. This result was consistent with the change in the residual strength shown in the stress–strain curve. The residual strength of the RCPB specimens decayed more slowly and exhibited the characteristic of “cracking but not breaking”, which means that they had good ductility. This was also the case because the toughness of the RCPB increased as the rubber content was increased.

It is also worth noting that the deformation capacity of the filler was increased due to the rubber’s better deformation capacity and ability to absorb elastic energy. In the process of being loaded, the filled body with rubber can store more elastic energy and, therefore, has better deformation capacity.

**Figure 14:**
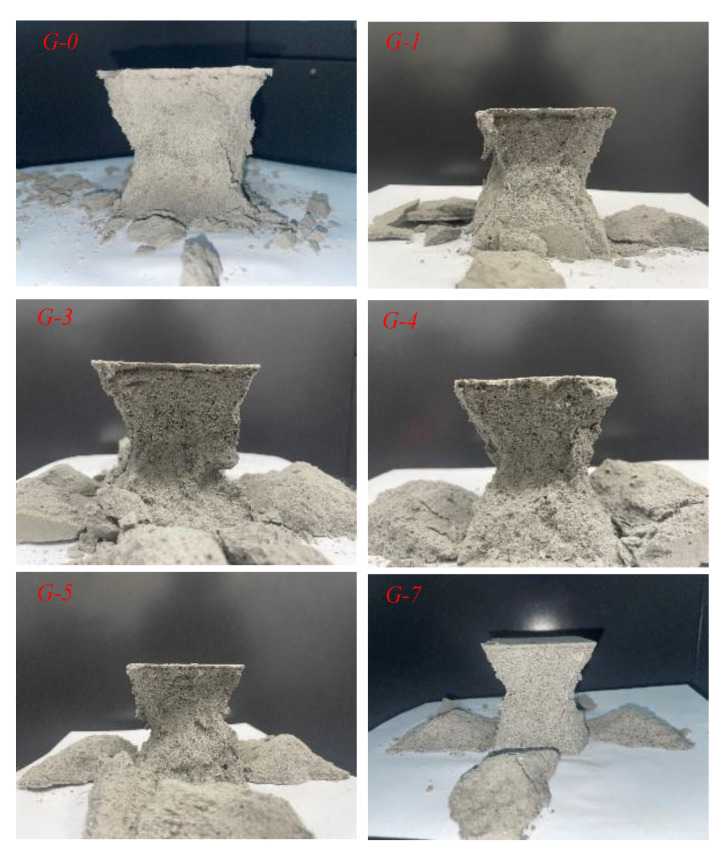
Morphologies of specimens after destruction. *G-1*~*G-7* represent the group to which the test block belongs, where the number represents the rubber content (%).

The morphologies of the specimens after destruction are shown in Figure 14. It can be seen here that the specimen without rubber mainly showed the characteristic of brittle damage. The specimens were broken into smaller pieces. The damaged area was concentrated in the specimens containing rubber, unlike in the specimen that did not contain rubber. As shown in Figure 14, the damaged RCPB specimens presented an “X” shape when viewed from four angles. The skeleton structure of the RCPB was not destroyed at this time, as it still had a certain load-bearing function, which is the reason for the high residual strength of RCPB. However, the overall strength of the specimen showed a downward trend because the cross-sectional area, bearing the load in the middle, was greatly reduced.

## 4. Conclusions

In the process of underground mining with adjacent field blast filling, the filling body can be damaged due to the dynamic impact from the blasts. Therefore, the impact resistance of the filling body is very important. Adding recycled rubber into the filler can not only increase the impact resistance of the filler, reduce the depletion rate of the ore, and save the cost of beneficiation, but it can also solve the environmental problems caused by waste rubber. In this paper, the failure process of RCPB is studied through physical tests. The influence of rubber on the UCS, peak strain, and toughness of RCPB was studied using UCS tests. The failure process of RCPB was analyzed by combining it with DIC technology. In addition, the relationship between the ultrasonic wave velocity and UCS was analyzed through UPV tests. The main conclusions of this study are as follows:

1. Although the UCS of RCPB is reduced as a result of the rubber, its peak strain and toughness are increased. The strength of the specimens in this study decreased by 15.7% after curing for 28 days, when the rubber content was increased from 0 to 4%. Moreover, the peak strain of the specimens increased by 82.5%, and the toughness of the specimens increased by 38%. This means that RCPB has better deformability, and the impact resistance of CPB can be increased with the addition of rubber. Increasing the impact resistance of RCPB can be achieved by designing its strength and toughness reasonably.
2. The UPV of RCPB decreases with an increase in its rubber content. There is an exponential function relationship between the UCS and the UPV of RCPB: the lower the wave speed, the lower the intensity. The main reason for this is that the UPV of rubber is relatively low. The propagation of the ultrasonic longitudinal wave in RCPB is also affected by the weak interface between the rubber and the filling matrix.
3. The fracture process of RCPB is divided into the following four main stages: the original crack compaction stage, the linear elastic stage, the plastic yield stage, and the post-peak failure stage. DIC technology was used to observe the local strain field of the specimens in this study. The stress–strain curve of the RCPB specimens were significantly different from the control specimen without rubber. The strain corresponding to the plastic-yielding stage increased as the rubber content increased. The ductility of RCPB was also greatly enhanced.
4. The specimens with added rubber mainly exhibited “X”-type failure. Since the rubber increased the toughness of the filled body, the filled body cracked but did not break. Furthermore, the residual strengths of the RCPB specimens were proportionally higher.

## Figures and Tables

**Table 1:** Ratio of materials.

Groups	Rubber (%)	Cement (%)	Tailings (%)	Water (%)
G-0	0.0	5.0	95.0	26.2
G-1	1.0	5.0	94.0	26.2
G-3	3.0	5.0	92.0	26.2
G-4	4.0	5.0	91.0	26.2
G-5	5.0	5.0	90.0	26.2
G-7	7.0	5.0	88.0	26.2

## Data Availability

Not applicable.
